# Role of Oxidative Damage in Alzheimer’s Disease and Neurodegeneration: From Pathogenic Mechanisms to Biomarker Discovery

**DOI:** 10.3390/antiox10091353

**Published:** 2021-08-26

**Authors:** Francesca Romana Buccellato, Marianna D’Anca, Chiara Fenoglio, Elio Scarpini, Daniela Galimberti

**Affiliations:** 1 Department of Biomedical, Surgical and Dental Sciences, University of Milan, 20122 Milan, Italy; elio.scarpini@policlinico.mi.it (E.S.); daniela.galimberti@policlinico.mi.it (D.G.); 2 Fondazione IRCSS ca’ Granda, Ospedale Policlinico, 20122 Milano, Italy; marianna.danca@policlinico.mi.it; 3 Department of Pathophysiology and Transplantation, University of Milan, 20122 Milan, Italy; chiara.fenoglio@unimi.it

**Keywords:** Alzheimer’s disease, oxidative damage, neurodegeneration, biomarker

## Abstract

Alzheimer’s disease (AD) is a neurodegenerative disorder accounting for over 50% of all dementia patients and representing a leading cause of death worldwide for the global ageing population. The lack of effective treatments for overt AD urges the discovery of biomarkers for early diagnosis, i.e., in subjects with mild cognitive impairment (MCI) or prodromal AD. The brain is exposed to oxidative stress as levels of reactive oxygen species (ROS) are increased, whereas cellular antioxidant defenses are decreased. Increased ROS levels can damage cellular structures or molecules, leading to protein, lipid, DNA, or RNA oxidation. Oxidative damage is involved in the molecular mechanisms which link the accumulation of amyloid-β and neurofibrillary tangles, containing hyperphosphorylated tau, to microglia response. In this scenario, microglia are thought to play a crucial role not only in the early events of AD pathogenesis but also in the progression of the disease. This review will focus on oxidative damage products as possible peripheral biomarkers in AD and in the preclinical phases of the disease. Particular attention will be paid to biological fluids such as blood, CSF, urine, and saliva, and potential future use of molecules contained in such body fluids for early differential diagnosis and monitoring the disease course. We will also review the role of oxidative damage and microglia in the pathogenesis of AD and, more broadly, in neurodegeneration.

## 1. Introduction

The longer life expectancy will lead to an increase in ageing-related neurodegenerative disease in the next future [1,2]. A rising challenge in the ageing population is the treatment and management of dementia patients, as public health care worldwide will face an increase in cases and the global cost of dementia patient treatment [3,4].

The most common cause of dementia is Alzheimer’s disease (AD), a progressive neurodegenerative disorder, affecting over 50 million people worldwide [4]. The disease is irreversible and presents with neurodegeneration caused by toxic aggregation of extracellular amyloid plaques, and intracellular neurofibrillary tangles of hyperphosphorylated tau protein [5]. Individuals with AD show memory loss, intellectual disabilities, changes in personality, and behavior. At present, acetylcholinesterase (AChE) inhibitors donepezil, galantamine, rivastigmine, and the N-Methyl-D-aspartate (NMDA) antagonist memantine are the only symptomatic medications approved by American and European regulatory agencies to treat AD [6]. In June 2021, the FDA gave a conditional approval of the first disease-modifying drug for the treatment of AD, aducanumab, a monoclonal antibody designed against the amyloid build-up, even if its efficacy has to be confirmed in large phase III–IV studies. In the hope that this treatment will be effective, the search for new potential treatments has never stopped and there is increasing interest in healthy food habits and alternative treatments which could prevent the cognitive decline or alleviate the memory loss in AD.

Although the aetiology of AD is still debated, the most studied pathogenic mechanism has been the “amyloid cascade hypothesis” proposed in 1991 by Hardy and Allsop, who suggested that inappropriate processing of amyloid-β (Aβ) precursor leads to the build-up of amyloid plaques, the formation of tau tangles, and ultimately to neuronal death [7].

About 2–3% of AD cases are due to autosomal dominant mutations. The rest are sporadic cases and are prevalent in the aging population [8]. Independently of genetic aspects, it is well known that AD pathogenesis starts decades before the symptoms appear. Following the new research framework (2018-NIA-AA-RF), AD is not defined by its clinical consequences but by its underlying pathology measured by lifetime biomarkers. Regardless of the presence of clinical symptoms, both Aβ and phosphorylated tau pathology are required for classification as AD, whereas Aβ deposition alone is an early sign, labelling the pathologic change towards AD [9]. In 2021, new recommendations for the clinical diagnosis of AD were proposed by the International working group, highlighting that the diagnosis of AD should be restricted to people who have positive biomarkers together with specific AD phenotypes. Cognitively unimpaired individuals who are only positive to biomarkers should be considered at-risk for progression to AD [10].

There are different pathogenic hypotheses to understanding AD aetiology and the early stages of AD [11]. The study of AD pathogenesis is significant not only for discovering new therapeutic targets and set up new clinical trials, but also to highlight potential biomarkers for the diagnosis of early stages of AD, in individuals with Mild Cognitive Impairment (MCI), or prodromal AD.

In AD, reactive microglia and astrocytes are associated with the amyloid plaques and respond to Aβ with the expression of pro-inflammatory cytokines [12,13,14]. Activated microglia are also observed close to tau protein in neurofibrillary tangles [12]. Further, activated microglia can respond to injured neurons in a neuroprotective manner and, afterwards, become toxic, contributing to neuroinflammation and neurodegeneration [15]. Microglia can respond to Aβ, or to other danger-associated molecules released by damaged cells, by producing reactive oxygen species (ROS) [16]. Thus, the overproduction of ROS and a decrease in antioxidant defenses can lead to oxidative stress (OS) [17]. The role of microglia in contributing to OS induced by ROS production and some of the molecular mechanisms implicated in ROS production will be analysed in this review.

Past and recent studies have demonstrated that oxidative damage is not only an early event in the pathogenesis of AD, occurring before the onset of clinical symptoms but also a factor contributing to the disease progression [17,18,19,20,21]. The role of oxidative damage in the pathogenesis of AD and more broadly in neurodegeneration will be discussed.

**Figure 1:**
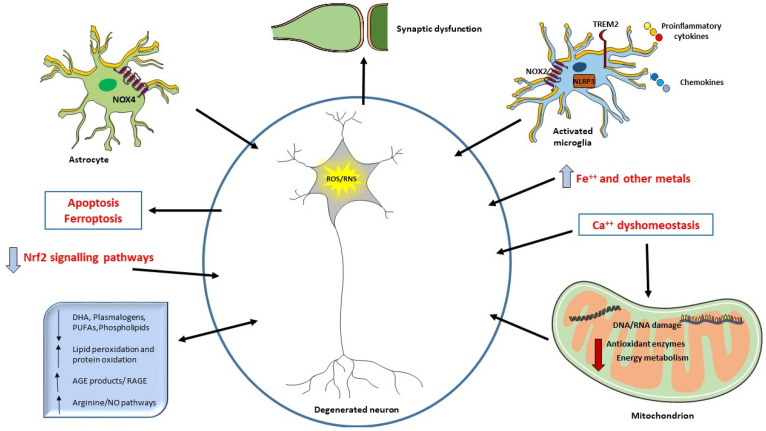
The neurodegeneration occurring in AD can be related to ROS overproduction and/or a decrease in antioxidant defenses. Aβ deposition can also be directly responsible for ROS increase when it interacts with microglial surface receptors or mitochondria. Mitochondria are the primary source of ROS but also a target of ROS and alteration in energy metabolism have been described in AD patients. ROS may also be considered a second messenger and induce different signaling pathways or the synthesis of antioxidant enzymes (Nrf2). Oxidative damage to mitochondrial DNA and RNA, proteins, and lipids are detected in AD brain. Increased levels of iron and other metals can be responsible of ROS production and iron has been related to ferroptosis. Ca^++^ influx alterations and the consequent mitochondrial dysfunction are also crucial in oxidative damage. Aβ can trigger NO production, an RNS. Oxidative damage affects synapses, causing cognitive impairment in AD. Decreased levels of different lipids have been correlated with cognitive deficit in AD, suggesting their use as biomarkers or in potential clinical intervention.

Oxidative damage products, as the ones deriving from oxidation of lipid, DNA, RNA, or glycation ends products, are extensively studied, and are suggested as biomarkers in neurodegeneration and AD [22,23,24]. They are detected in body fluids such as blood, plasma, serum, cerebrospinal fluid (CSF), urine, and saliva [25,26,27]. The availability of such molecules in peripheral circulation and their use as potential biomarkers of AD is of paramount importance, overtaking the invasiveness and cost of the usual diagnostic and imaging tools. See Figure 1 for summary of the interplay between ROS, oxidative damage products, and cellular components in AD.

In this review, we will discuss the current knowledge on oxidative damage products. In addition, we will analyze the potential use of this body of knowledge to identify sensitive and reliable biomarkers in AD and in the early stages of the disease as MCI and prodromal AD. Recent data from metabolomic and proteomic studies can help to identify the potential biomarker [22,27]. The ideal biomarker should permit a diagnosis before the clinical onset of the disease and correlate with neuropathological findings and/or the progression of the disease.

## 2. Products of Oxidative Damage in AD

OS is defined by the disruption of the equilibrium between ROS and/or reactive nitrogen species (RNS) levels and antioxidants defenses [18]. Increased levels of ROS and/or RNS together with decreased levels of antioxidants can lead to oxidative damage to cells, molecules, and biological systems [18]. In the brains of individuals with AD and preclinical AD (i.e., evidence of Aβ and tau deposition in the brain without any clinical manifestation of the disease), as well as patients with MCI (a mild clinical impairment with no proof of underlying pathology with biomarkers), many oxidative damage products are measured, and numerous investigations have pointed to OS as a key and early player in the pathogenesis of neurodegeneration and AD [18,19].

The brain is very susceptible to OS because it is rich in polyunsaturated fatty acids, has a high oxygen consumption and a high content of metals in particular, iron, that can catalyze oxidative reactions, in contrast, it has less antioxidant enzymes in comparison with other organs [28]. The result of the reaction of ROS with different substrates can be protein, nucleic acid oxidation or lipid peroxidation. All of these markers of OS have been described as an early alteration in the AD brain, and this concept has been used in the past to support the ‘oxidative stress hypothesis’ in the pathogenesis of AD [20,28,29,30]. The oxidative stress hypothesis can be applied to other neurodegenerative diseases, such as Parkinson’s Disease (PD) and Amyotrophic Lateral Sclerosis (ALS) where the accumulation of oxidative damage over time leads to the late life onset and to the progressive nature of these diseases [29]. It has been hypothesized that tissue injury, aging, ischemia, increased metal levels or energy metabolism deficit can induce ROS generation independently from Aβ deposition [29]. Even if ROS generation is secondary to other initiating causes, it plays an important role in the events that lead to neuron death in AD [29].

3-Nitrotyrosine is a product of oxidative damage on proteins generated when a superoxide radical reacts with nitric oxide (NO) to form peroxynitrite, which cause nitration of proteins on tyrosine residues. 3-nitrotyrosine and protein carbonyls, these last produced mainly by the action of free radicals on the peptide chain or by oxidation of amino acids, are used as biomarkers of oxidative damage in the brain of AD and MCI [18,24,31,32,33]. NO, also classified as an RNS, is synthesized from L-arginine by three different isoforms of the enzyme NO synthase (NOS), i.e., endothelial (eNOS), neuronal (nNOS), and inducible (iNOS) which catalyze a two-step oxidation of L-arginine to NO and L-citrulline [34,35]. NO is a gaseous molecule, with a lone pair of electrons which can easily diffuse across membranes acting as a vasodilator, neuromodulator, and inflammatory mediator [34]. At high concentration, NO reacts with superoxide radical and generates peroxynitrite, contributing to OS. iNOS is implicated in the pathophysiology of neurodegenerative diseases including AD, as Aβ can trigger NO production with an unknown mechanism, which leads to increased levels of free radicals, mitochondrial damage, and consequently neuronal function alterations [34]. Due to its gaseous nature and its short half-life, it is difficult to measure NO as a marker of OS, but its metabolites in the arginine/NO pathway such as L-arginine, asymmetric and symmetric dimethylarginine, and dimethylamine have been studied in metabolomic analysis. Untargeted and targeted metabolomics studies have indicated that L-arginine/nitric oxide (NO) pathway is altered in AD [35,36].

Membrane phospholipids contain arachidonic and docosahexaenoic (DHA) acids, which are abundant in the brain and vulnerable to ROS attack. Decreased levels of phosphatidylcholine, phosphatidylethanolamine and the phospholipid precursors, choline and ethanolamine, have been described in AD and are considered specific for the pathogenic mechanism of AD. The depletion of phospholipids in AD has been referred to an increase of ROS-mediated lipid peroxidation [29]. 4-hydroxy-2-trans-nonenal (HNE) is produced in the brain via lipid peroxidation of arachidonic acid present in neuronal membranes [37]. HNE could be considered an apoptotic inducer because of its ability to spread OS and form protein adducts involved in neurodegenerative diseases [38]. This highly reactive aldehyde and also hexenal and 2-propene-1-al (acrolein) can react with specific amino acid residues on proteins altering the conformation and functions of such proteins. Increased levels of HNE are observed in AD and MCI brains [24,38]. Neurofilaments are a primary target of HNE as HNE-adducts to neurofilaments have been found [38]. Further, HNE have been detected in Aβ plaques and in the cerebrospinal fluid in AD patients [39,40]. By altering membranes, HNE could impair ionic and energetic metabolism and cause neuronal cell death [37].

Redox proteomic studies confirm previous results that carbonylated proteins are present in CSF of amnestic MCI patients compared with controls, and these proteins remain oxidized in the progression towards AD [40]. F2-, F3- F4-isoprostanes are prostaglandin isomers also produced by ROS attack on arachidonic acid in membrane phospholipids. Analysis of specific isoprostane may reflect increased OS in AD [41].

Advanced glycation end products (AGE) are irreversible products of the glycosylation of lysine or arginine residues of proteins and are considered biomarkers of oxidative damage. The impact of AGE adducts on various aging related diseases including AD, the role of increased oxidative stress in promoting the production of AGE-related adducts and the consequences in aging-related diseases are well documented in the review of Rungratanawanich et al. (2021) [42]. In the brain, OS can cause the production of AGEs and OS can also be generated due to AGEs formation in this organ. This vicious cycle may increase oxidative damage, leading to the initiation and progression of neurodegenerative diseases. AGEs and the receptor for advanced glycation end-products (RAGE) can play an important role in AD pathogenesis as higher levels of RAGE expression in neuronal, microglial, and endothelial cells in patients with AD compared with age-matched, non- demented controls are found [43,44,45,46]. The levels of expression of RAGE are correlated to the severity of the disease indicated by clinical score of the amyloid plaque or tangle [45,47]. Therefore, one of the most abundant AGE-protein products in the brain is the AGE-albumin adduct, which causes RAGE overexpression in primary neurons from human AD brains. OS and Aβ aggregation increase the formation of AGE-albumin adduct [48]. Aβ can also bind RAGEs to activate the NF-κB, and other signal transducers and activators of transcription pathways, leading to neuronal cell death and neurodegeneration [49].

Oxidation of DNA and RNA produced by ROS also occurs in AD [50,51,52,53]. 8-Hydroxy-deoxyguanosine (8-OHdG) is the product of oxidative DNA damage while 8-oxo-guanine (8-oxoGua), and 8-oxo-guanosine (8-oxoGuo) are markers of oxidative RNA damage. Both these oxidative damage products are the most commonly measured biomarkers of OS. Oxidative RNA damage can affect not only mRNAs but also non-coding RNA species [54]. Dysregulation of microRNAs (miRNAs) has been implicated in neurodegenerative disorders such as AD and this matter is reviewed by Nunomura and Perry (2020). Further, an in vitro study has demonstrated that miRNAs can be directly oxidized and can misrecognize mRNAs that are not their original targets [55]. OS can also affect the expression of multiple miRNAs, which regulate genes involved in the OS response in neurodegeneration in AD, PD, ALS, and Huntington’s disease (HD) [56,57].

The brain iron is stored within the ferritin protein complex, preventing iron from converting hydrogen peroxide into a highly toxic hydroxyl free radical. Through the production of these ROS, iron can induce severe brain damage [28,58]. Iron and ferritin are described to be associated with amyloid deposition and microglia [59,60]. Their levels are elevated in neuronal tissue and in the amyloid plaques [61]. Alongside the capacity of iron to induce OS alone catalyzing the formation of ROS, iron can bind histidine residues of Aβ and in this way trigger the redox activity of the iron-Aβ aggregate [62].

## 3. Mitochondria as Sources of ROS and Target of Oxidative Damage

In the AD brain, mitochondria can be sources of ROS and also the target of OS, especially mitochondrial DNA (mtDNA) is susceptible to oxidative damage [19,53,63]. It is demonstrated that there is a progressive age-related accumulation of oxidative damage to DNA in the human brain and that mtDNA is preferentially affected. The increased amount of 8-OHdG in mtDNA and nuclear DNA is demonstrated in the aging human brain and in AD [50,51,64]. Hirai et al. have demonstrated that oxidative damage marked by 8-OHdG and nitrotyrosine occurs in the neurons of AD patients. Morphometric analysis showed a reduction of mitochondria in these same neurons and an accumulation of mtDNA and mitochondrial proteins [53]. These abnormalities are found in neurons lacking neurofibrillary tangles, suggesting that mitochondrial abnormalities are one of the earliest pathological changes in AD. These results confirmed that mtDNA is a target of oxidative damage and that the increased damage to DNA contributes to AD and the neurodegenerative process [51,53].

The studies on oxidative-associated damage on mitochondria in AD have sustained the “mitochondrial cascade hypothesis” of AD pathogenesis, alongside the classical amyloid cascade hypothesis [65]. In this explanation, the mitochondrial function declines with age and leads to increased production of ROS [65,66]. Besides, amyloid oligomers can access mitochondria, contributing to compromise their function and triggering a vicious circle in which it is not defined if the accumulation of Aβ came before mitochondrial declined function or after [67].

Dysregulation of Ca^2+^ homeostasis has also been reported as cause of mitochondrial-induced production of ROS in AD [68,69]. Aβ can be toxic on neuronal mitochondria and causes Ca^2+^ elevation, changes in mitochondrial size and shape in APP/PS1 AD mouse model [70]. Excessive Ca^2+^ accumulation into mitochondria leads to decreased mitochondrial membrane potential and increased ROS production in the same AD mouse model [67,68,70]. In this way the toxic action exerted by Aβ causes mitochondrial fragmentation that has been reported to precede neuronal cell death [71,72]. The maintenance of a healthy mitochondrial pool is essential for neuronal fitness, indeed dysfunctional mitochondria are degraded through selective autophagy, a mechanism called mitophagy. Dysregulated mitophagy has also been implicated in neurodegenerative diseases, as PD and AD, as well as aging [73]. Excessive calcium influx induced by overstimulation of the NMDA receptor is the mechanism implicated in the neuroprotective effect of Memantine. Memantine is a drug, approved in US and in EU, for the treatment of the progressive symptomatic decline in patients with moderate to severe AD [74]. The drug exerts its neuroprotective effect blocking NMDA Receptor (NMDAR). Aβ oligomers can target and activate NMDAR leading to a rapid increase in neuronal calcium levels and ROS production in mature hippocampal neurons in culture [75]. During pathological activation of the NMDAR, memantine blocks excessive calcium entry through the channel and Aβ-induced OS, providing a specific biological mechanism for the therapeutic action of memantine.

Recently, sirtuin 3 (SIRT3) defects have been considered to contribute to OS in AD mitochondria. Indeed, SIRT3 has been linked to dysregulation of mitochondrial DNA expression, ROS accumulation and neuronal damage in AD [76,77]. The importance of SIRT3 is highlighted in this report as SIRT3 has been found exclusively in mitochondria and its function is to eliminate reactive oxygen species and inhibit apoptosis [76,77].

Glucose and energy metabolism can be affected by the oxidative damage to the mitochondria. Glucose metabolism is dysregulated in AD and MCI and the metabolic rate of glucose is decreased in the brain of patients with senile dementia [18]. De Leon in a longitudinal FDG-PET study prognosticated the future cognitive decline and MCI among normal elderly individuals using decreased glucose metabolism as a predictor of the clinical change [78]. Using redox proteomics to analyze the protein profile in brain tissue samples of patients with AD, Di Domenico and Butterfield suggest that in affected brain areas, oxidative modification of the glycolytic enzyme aldolase, triosephosphate isomerase, glyceraldehyde- 3-phosphate dehydrogenase, phosphoglycerate mutase 1, and α-enolase occurs. In addition, oxidative modifications to enzymes involved in TCA cycle, ATP production in brain mitochondria are described in the brains of individuals with MCI and AD [24]. Large scale analysis of proteomic in AD brains and CSF demonstrated that activation of microglia and astrocytes are associated with energy metabolism [27]. This activation may be neuroprotective and anti-inflammatory in preventing the progression to AD. Increased levels of glucose, carbohydrate, and protein metabolism in CSF of patients with AD were also observed [27]. Taken together, these results suggest the link between energy metabolism and OS in AD. Glucose metabolism, ATP production, and OS are deeply linked in the energy metabolism of the cell, while mitochondria are the cellular organelles deputed to control the energy metabolism. Deficiency in energy metabolism deriving from one or more levels can represent a key role in the pathogenesis of AD.

## 4. Neurodegeneration and OS

AD neuropathology is characterized, macroscopically, by cerebral cortical thinning and atrophy where synapse and neuronal loss contribute to the atrophy. From a microscopic point of view, the hallmarks of the pathology of AD are neuritic amyloid plaques and neurofibrillary tangles. The alternative “Aβ oligomer hypothesis”, supported by in vitro, in vivo, and ex vivo models, points to the toxic Aβ oligomers (AβO)s rather than amyloid plaques as key players in AD pathogenesis [79,80,81]. (AβO)s are considered the most toxic and pathogenic form of Aβ [79,82]. In this hypothesis, after cleavage from the membrane, Aβ peptides aggregate to form AβOs. Part of the oligomers which are not going to form fibrils may induce the neuron damage responsible for cognitive decline and dementia. The collective body of evidence, reviewed by Cline et al., supports a pathogenic mechanism in which AD neuropathology and cognitive loss are the consequences of the AβOs toxicity on neurons. Blocking AβOs or AβO receptor has been considered a disease-modifying treatment in AD. Indeed, Aducanumab has been designed to target AβOs and fibrils to slow the cognitive decline. Additionally, RAGE has been identified as an AβO targeted receptor and Azeliragon, an inhibitor of RAGE, is in phase 3 clinical trial as the therapy shows significant delay in AD cognitive decline. The mechanism of toxicity of AβOs can be exerted by binding specific receptors and activate receptor transduction mechanism, by a direct interaction with different cells as neurons, microglia, astrocytes, or organelles such as mitochondria [83,84]. As discussed before, mitochondria respond to the toxic action of AβOs producing ROS, which can mediate apoptosis of the neuronal cells [69,82].

Cognitive deficits in MCI patients and decreased executive and reasoning abilities in AD are caused by the oxidative damage of small Aβ oligomers to the synaptic membranes [83,84,85]. In vivo and in vitro studies support a direct relationship between OS and synaptic dysfunction in AD [18,86,87,88,89,90].

The Aβ oligomer hypothesis sustains another emerging knowledge: the cellular and molecular basis for neuroanatomic selectivity seen in the AD, and the neuron to neuron spread of the toxic Aβ protein [79,91,92]. Indeed, the progression of AD pathology correlates to the diffusion of amyloid deposition to specific neurological structures, following this regional and temporal pattern is possible to identify specific stages in the progression of the disease. The staging of the progression was postulated by Braak [93]. In this regard, it is possible to hypothesize regional and temporal differences between the different neuronal population responses to OS. In this view, entorhinal cortex (EC) neurons are particularly prone to damage by OS and mitochondria dysfunction [94,95]. The EC is a vital component of the medial temporal lobe, contributing, during pathological condition, to downstream changes in its afferent region, the hippocampus. Neurodegeneration in the EC and hippocampus has been clearly linked to impairments in memory and cognitive function in the early phases of AD [91,96]. Oxidative damage to RNA in EC, hippocampus, subiculum, and temporal neocortex of subjects with AD has been described. The levels of 8-OHdG increase early before pathological changes occur, and this can explain that the selective vulnerability of neurons in the EC during AD may be related to the vulnerability of these particular neurons to oxidative damage [19].

Iron levels in the hippocampus of AD patients, are significantly elevated [97]. Abnormalities in iron homeostasis in brain tissue can increase ROS production, causing noxious oxidative damage to sensitive cellular structures, and trigger a new cell death process called ferroptosis [98,99]. There is still not clear evidence to relate ferroptosis to neurodegeneration occurring in AD, but several studies have considered targeting iron metabolism, using iron chelators, as a potential drug in the treatment of AD [100] (Deferiprone clinical trial, 3D Study NCT03234686). Ferroptosis-dependent cytotoxicity induced by the activation of OS has also been described in human astrocytes from AD patients [101].

## 5. Microglia and Astrocytes as Sources of ROS in AD

Colocalization of reactive microglia with Aβ deposits is a hallmark of AD pathology and has been reported also in brains from AD mouse models [102,103]. Microglia express pattern recognition receptors (PRRs), innate immune cell receptors that respond to danger- (DAMP) or pathogen-associated molecular patterns [102,103]. The nucleotide-binding-like receptor (NLR) belongs to PRRs and can trigger the formation of multiprotein complexes, called inflammasome [104]. NLRP3 inflammasome is expressed exclusively by microglia in the CNS and may detect noxious agents or disturbances in the cellular environment [104]. Various studies have described a role of NLRP3 signaling in different neurologic disorders, including multiple sclerosis (MS), ALS, prion diseases, and AD [105,106]. In AD, Aβ can act as DAMP and its interaction with microglia creates an oxidative and neuroinflammatory environment through an increased production of ROS and release of proinflammatory cytokines [107]. Further, NLRP3 activity is associated with impaired clearance of Aβ by microglia [104].

NADPH oxidase (NOX) belongs to a group of ROS generating enzymes that can be activated by different stimuli in response to neuronal injury, inflammation, or OS. NOX activation is evident in the AD brain, in addition NOX2 is highly expressed in microglia [108,109]. In this scenario, activated microglia might contribute, through the activation of NOX2, to trigger or maintain the OS in AD. NO X4 is the astrocytes isoform, and its levels are reported significantly elevated in astrocytes of patients with AD and APP/PS1 double-transgenic mouse model of AD [101]. The levels of two products of oxidative damage, 4-HNE and MDA, were also significantly elevated, suggesting that the elevation of NOX4 promotes the impairment of mitochondrial metabolism, mitochondrial ROS production and fragmentation in human astrocytes [101]. NOX4-mediated ferroptosis is also described in human astrocytes in this experimental setting [101].

ROS produced by activated microglia can also act as a second messenger, inducing different signaling pathways. Zhang and colleagues (2016) describe several cellular signaling pathways that ROS can induce [110]. In particular, the transcription factor nuclear factor-erythroid 2 p45-related factor 2 (Nrf2) is involved in the cellular redox metabolism, protecting cells against OS by binding to antioxidant response elements (ARE) in the promoters of antioxidant genes. The trigger of the Keap1-Nrf2-ARE signaling pathway is responsible for inducing a protective mechanism against OS in many diseases including AD and PD [111,112]. The increased level of intracellular ROS promotes the dissociation of Nrf2 and Keap1. Then, dissociated Nrf2 is transferred to the nucleus where it targets genes encoding for detoxification enzymes such as glutathione synthetase (GSS), glutathione reductase (GR), thioredoxin (TRX), thioredoxin reductase (TRR), and peroxiredoxin (PRX) to prevent the OS [110]. Downregulation of Nrf2-target genes have been reported in AD, and other neurodegenerative conditions [112]. Nrf-2 activation can occur not only in response to OS but can be induced by plant extracts or synthetic compounds with antioxidant properties [113]. The Nrf2 activating compound dimethyl fumarate is an approved FDA-therapy for MS and clinical trial is underway for its use in Friedrich’s ataxia. Another Nrf2 activating compound is *Centella asiatica* extracts, which can activate Nrf2 in neuroblastoma cells, isolated primary neurons and in the brains of AD and aging mouse models. This activation improved mitochondrial and cognitive function, and enhanced synaptic density [113,114,115].

Proliferation and activation of microglia in the brain, around amyloid plaques, is a prominent characteristic of AD [102]. In the attempt of digesting Aβ, microglia can release not only ROS but also pro-inflammatory cytokines or chemokines to recruit other cells. Anti-inflammatory cytokines, then, modulate phagocytosis to limit the process [116]. In this way, activated microglia can contribute to neuroinflammation and neuronal cell death. Indeed, chemokines and cytokines increased levels in plasma and CSF are a very early event in AD pathogenesis, even preceding the clinical onset of the disease, as demonstrated by subjects with MCI who developed AD over time, representing a crucial step in the progression of the disease [117,118,119].

Recent findings highlight the potential involvement of the triggering receptor expressed on myeloid cells 2 (TREM2) in AD pathology, neurodegeneration, and neuroinflammation. In the brain, TREM2 is exclusively expressed by microglia and its function is to maintain microglial health during stress events and to enable the progression of microglia towards the profile of disease-associated microglia (DAM) [120]. TREM2 facilitate microglial phagocytosis and clearance of Aβ and apoptotic neurons [121]. The mutation in TREM2, has been associated with a three-fold higher risk to develop AD [122]. Regarding the relation of TREM2 with OS, it has been supposed that a down-regulation of microglial TREM2 expression and signaling might be one of the major pathogenetic mechanisms in sporadic cases of AD in which advanced age, OS, neuroinflammation contribute to the suppression of wild-type TREM2, considering that TREM2 mutation is rare in humans [123].

## 6. Focus on Experimental Models of AD and Potential Antioxidant Therapeutic Targets

The pioneering studies, conducted to validate the early changes of products of oxidative damage, found in specimens of AD patients, in a transgenic mouse model (Tg2576) of AD amyloidosis, dated back more than 20 years [20,124]. In the last 20 years several other experimental models are designed to reproduce the hallmarks of the pathology of AD and to study products of oxidative damage in animal models.

Some of the molecular mechanisms of the pathogenesis we have revised in the present review are studied in these models. To counterbalance the increase of OS that is characteristic in age-related neurodegenerative diseases antioxidant treatment are studied in experimental models and in human trials.

While some of the studies of early biomarkers focused on wide-proteomic analysis to find a reliable and specific marker of OS in human fluids, the molecular mechanisms to justify the involvement of mitochondria, microglia and the use of antioxidant pathway as therapeutic target in AD are better analyzed in animal experimental models or in-vitro models. In the recent literature we can find significant trends in the new approaches to study the role of oxidative damage in AD. Indirect proofs of the role of oxidative damage derive from the studies of biochemical/molecular mechanisms, which minimize the accumulation of such deleterious products in experimental models of AD.

To maintain genome integrity, the misincorporation of oxidized nucleotides is prevented by various repair enzymes, such as human MutT homolog 1 (MTH1). MTH1 hydrolyzes 8-oxo-dGTP to its corresponding monophosphates, 8-oxo-dGMP, avoiding 8-oxo-dG incorporation into DNA. Meanwhile, 8-OxoG DNA glycosylase-1 (OGG1) excises 8-oxoG paired with cytosine in DNA, minimizing 8-oxoG accumulation in DNA [125]. Oka et al., studied the role of 8-oxoG accumulation in the pathogenesis of AD, utilizing a knockout of Mth1 and Ogg1 genes in a 3xTg-AD background. They demonstrated that MTH1 and OGG1 deficiency increased 8-oxoG accumulation in nuclear and mitochondrial genomes, causing microglial activation and neuronal loss with impaired cognitive function at 4–5 months of age. The use of minocycline, an inhibitor of microglial activation and neuroinflammation, in this model decreased the nuclear accumulation of 8-oxoG in microglial cells, and inhibited microgliosis and neuronal loss. Further, gene expression profiling revealed that MTH1 and OGG1 efficiently suppress progression of AD by inducing various protective genes in 3xTg-AD brain. These results highlighted that the potential suppression of 8-oxoG accumulation in brain genomes could be a new approach for the prevention and treatment of AD [125].

On the same tune, the report of Pao et al. 2021 shows that histone deacetylase (HDAC)1 modulates OGG1-initated 8-oxoguanine (8-oxoG) repair in the brain [126]. HDACs enzymes remove acetyl groups from lysine residues of histones and non-histone proteins modulating transcription, chromatin remodeling, and DNA repair. Deregulation of HDAC1 induces alteration in cell cycle activity and DNA damage leading to neuronal death in cultured neurons and mouse brain [127]. Further, sirtuin (SIRT) 1 can also bind the class I histone deacetylase HDAC1, deacetylate HDAC1 and stimulate its enzymatic activity [128]. HDAC1-deficient mice show DNA damage accumulation and cognitive impairment during aging [126]. Further, this work showed elevated 8-oxoG along with reduced HDAC1 activity and downregulation of a gene set critical for brain function in the 5XFAD mouse model of AD, uncovering important roles for HDAC1 in 8-oxoG repair and highlights the therapeutic potential of HDAC1 activation to counteract the functional decline in brain aging and neurodegeneration [126].

AD pathogenesis has been linked to mitochondria as their health is essential in maintaining energy homeostasis and in preventing neuronal dysfunction. Stojakovic et al. observed that mitochondrial respiratory chain complex I might be a therapeutic target in AD [129]. Chronic treatment with complex I inhibitor, the tricyclic pyrone (CP2), which can penetrate the blood brain barrier (BBB) and accumulate in mitochondria, improves cognitive and motor function in transgenic mice, expressing a form of the amyloid precursor protein (APP) and presenilin 1 that leads to early onset AD (APP/PS1). Further, this treatment can reduce total Aβ levels triggering autophagy, one of the neuroprotective pathways essential for Aβ clearance. Taken together, the data suggest that CP2 treatment induces multiple protective mechanisms including autophagy, anti-inflammatory, and antioxidant responses, which contribute to reduce Aβ pathology [129].

Mitochondrial Tu translation elongation factor (TUFM or EF-Tu) can maintain mitochondrial respiratory chain activity and protect cell against OS. TUFM protein level is decreased in the hippocampus and cortex in the aged APP/PS1 mice [130]. Further, cellular ROS levels were affected by TUFM expression, and TUFM-mediated regulation of apoptosis and Tau phosphorylation is counteracted by the treatment with TEMPO, a potent antioxidant drug. Collectively, TUFM protein levels were decreased in APP/PS1 mice. Taken together these results pointed at TUFM involvement in AD pathology by regulating BACE1 translation, apoptosis, and Tau phosphorylation, in which ROS plays an important role [130].

Regarding the role of mitochondria and Aβ oligomers in AD pathology, Takeda and collaborators have studied the role of mitochondrial ubiquitin ligase, as MITOL is an integral mitochondrial outer membrane protein which regulates mitochondrial functions and morphology [131]. It is supposed to be downregulated in patients with AD [131,132]. MITOL-deleted APP/PS1 mice show severe cognitive impairment, synapse alteration, and neuroinflammation mediated by excessive generation of toxic and dispersible Aβ oligomers. It is possible to affirm that MITOL deletion triggers mitochondrial impairments and exacerbates cognitive decline in APP/PS1 mouse model with AD [131].

Glutathione (GSH) is a cellular antioxidant, and its depletion has been observed in AD [133]. The effect of dietary supplementation with γ-glutamylcysteine (γ-GC), the immediate precursor of GSH biosynthesis has been studied in the brains of APP/PS1 mice [134]. The authors report that the diet with γ-GC lowered the levels of brain lipid peroxidation, protein carbonyls and apoptosis, and maintain antioxidant status in APP/PS1 mice. Aβ pathology was reduced, and AChE activity was improved in APP/S1 mice on the γ-GC diet compared to APP/PS1 mice fed a standard chow diet. γ-GC may also lower inflammation and enhance Aβ plaque clearance in vivo as suggested by cytokines and matrix metalloproteinase levels in the brain. Supplementation with γ-GC improve learning and memory in this AD animal model as determined by their performance in Morris water maze [134].

The intranasal administration of a nano-formulation of graphene oxide loaded with dauricine has been studied in a mouse model of AD for its capability of protecting against oxidative damage induced by Aβ. This report highlights also the potential of non-invasive drug delivery system based on nanoparticles that can pass the BBB and reach the brain [135].

## 7. Products of Oxidative Damage in Blood, CSF and Other Body Fluids of AD Patients

The amyloid/tau/neurodegeneration (AT(N)) classification is used to divide biomarkers into those measuring β-amyloid (Aβ) deposition (A) (CSF Aβ levels or Aβ-positron emission tomography (PET)), pathologic phosphorylated tau (T) (CSF phospho-tau (p-tau) levels or tau-PET), and neurodegeneration (N) (18F-fluorodeoxyglucose-PET (FDG-PET), magnetic resonance imaging (MRI), or CSF total tau (t- tau) levels) [10,136]. These biomarkers are required for the biological diagnosis of AD. Blood-based biomarkers are not available for AD and the potential ones are derived from Aβ and Tau [137]. A non-invasive, peripheral approach of screening would be useful and the potential use of products of oxidative damage as markers in the early stages of the disease has been extensively studied. Although the brain is the most affected in AD the search for markers of OS on peripheral fluids has been always active (Table 1).

Few studies have analyzed the potential of urine, the most peripheral and available human fluid as a source of biomarkers in AD. The largest metabolic analysis performed on urine of AD and MCI patients have permitted to shape a urinary metabolic phenotyping. Urine contains metabolites that reflect a response to injury, including OS, occurring at high distance or carry information originated from the gut microbiome, a novel area of research in neurodegeneration and AD [138]. The strength of this report, even if not specific on oxidative markers, lies in the potential to shape a metabolic profile associated with AD and to correlate such metabolites to genetic variants [138]. Peña-Bautista and colleagues have recently developed some analytical methods to determine a panel of lipid peroxidation biomarkers in urine, plasma, and saliva samples [25,138,139,140,141,142,143,144]. Saliva is also considered a promising source of biomarkers which measure products of oxidative damage. These potential neurodegenerative biomarkers could represent a promising screening test for minimally invasive AD diagnosis and for monitoring the progression of the diseases. A very recent study of this group has evaluated lipid peroxidation compounds in preclinical AD patients and healthy elderly individuals [142].

Recently, the plasma lipidome signature has been proposed as a potential biomarker of AD. Liu and colleagues (2021) have examined the differences in plasma lipidome between AD patients and healthy age-matched controls and compared the lipid profiles to identify specific alterations in lipid metabolism [145]. Decreased levels of ethanolamine plasmalogens (PlsEtns) have been found to correlate with the severity of AD. However, the potential use of signature species of PlsEtns as biomarkers in AD as well as their potential in therapy have yet to be explored [146].

OS and inflammation can perturbate the high-density lipoprotein (HDL) proteome, possibly playing a role in AD pathogenesis. Loss of HDL-associated proteins with antioxidant action such apolipoprotein phospholipase A2, glutathione peroxidase-3, and paraoxonase (PON)-1 and -3 are supposed to be related to the early stage of AD. PON-3 modulating factors should be considered of interest in the study of potential AD treatments [147]. Additionally, Apolipoprotein A-I levels were decreased in serum of AD patients [148]

Salivary levels of AGE have been described to correlate with the deterioration in mini mental state examination (MMSE) score and have been suggested as potentially useful in the diagnosis of dementia [26].

AD can modify a number of metabolites which, even if not considered a product of oxidative damage, can be related to OS pathways. Serum metabolome analysis can dissect the metabolic heterogeneity in AD pathology and can associate specific metabolic alterations to AD phenotypes, permitting a more precise and personalized medicine. Regarding this experimental approach, several studies have been done. Arnold et al. (2020) highlighted the sex-specific dysregulations of energy metabolism, energy homeostasis, and (metabolic/nutrient) stress response, presenting the first evidence and molecular readouts for sex-related metabolic differences in AD [149]. The metabolic alterations described in this work suggest that females experience greater impairment of mitochondrial energy production than males [149]. A metabolomic study using high-resolution mass spectrometry with liquid chromatography conducted on CSF from normal controls and MCI revealed a metabolic signature characterized by impairments in sugars, methionine, and tyrosine regulation in MCI [150]. Blood metabolic signature revealed decreased levels of specific amino acids and lipids levels in AD, and three markers of oxidative potentially predictive in demarcating AD patients from control subjects: the isoprostane-pathway derivatives 8,12-iPF-2a IV, and PGD2, and the nitro-fatty acid NO2-aLA (C18:3) [22].

As previously discussed, NO is an RNS, and is implicated in OS in various neurodegenerative diseases. Untargeted and targeted metabolomics studies have indicated that L-arginine/NO pathway is altered in AD [35,36]. Further, using targeted metabolomics, Fleszar and colleagues have assessed L-arginine, L-citrulline, dimethylamine, asymmetric dimethyl arginine, and symmetric dimethylarginine in blood samples from AD, mixed-type dementia, and non-demented individuals and they demonstrated that L-Arginine/NO pathway is altered in neurodegenerative and vascular dementia in association with neurodegenerative and vascular markers of brain damage and severity of cognitive impairment [35].

Protein profiling approach is considered a promising strategy to assess pathological changes in both asymptomatic AD and later stages of the disease [151]. In this approach, proteomic data collected from postmortem brain tissue of control, asymptomatic AD, and AD cases have been organized in groups or modules with biological significance in underlying the disease and correlated with clinical features of AD [147]. Alterations in these disease-associated modules are strongly conserved across AD cohorts [151]. The largest proteomic study allowed to identify a protein network module linked to sugar metabolism as one of the modules most significantly associated with AD pathology and cognitive impairment [27]. Proteins from this module were elevated in CSF in early stages of the disease [27]. In this case, no specific protein has been linked to OS, but a group of proteins involved in one of the molecular mechanisms were potentially dysregulated during OS in AD. Obviously, the potential availability of blood-based biomarkers would overtake the invasiveness and cost of the usual diagnostic tools. Recently, a large-scale proteomic profile of human serum specimens has revealed consistent mitochondrial protein changes between control and AD samples [152]. Several redox markers were found altered in whole blood cells from AD patients, some of them (GR, GSH, GSSG, and GSSG/GSH) are already altered in MCI patients, suggesting their potential use as markers of prodromal AD. Further, they are associated with MMSE scores and seem to be useful to monitor cognitive decline in AD progression [153].

## 8. Antioxidants and Other OS Counteracting Drugs as Potential Therapy in AD

According to the definition, not only an increase in ROS levels but also a decrease in antioxidants levels can lead to OS. In response to OS in the brain, a number of protective mechanisms are up regulated to maintain the oxidative balance, in particular compensatory induction of antioxidant enzymes such as superoxide dismutase, glutathione reductase, catalase have been found in vulnerable neurons in AD [31]. Total antioxidant capacity (TAC) (including glutathione, ascorbic acid, uric acid, and bilirubin) is measured in plasma samples from AD and MCI patients and has been found depleted [117]. In addition, several nonenzymatic antioxidants such as vitamin C, vitamin A, vitamin E, and carotenoids were lower in the plasma of patients with AD compared with controls and are associated to increased levels of oxidative damage markers [155]. Reduction of antioxidants such as glutathione, glutathione peroxidase, glutathione-S-transferase, and superoxide dismutase are demonstrated post-mortem in the mitochondrial and synaptosomal fractions in the frontal cortex of MCI and AD patients [31].

Oxidative damage and antioxidant response were assessed in neurons and astrocytes of post-mortem frontal cortices of AD and non-demented individuals with Alzheimer’s Neuropathology (NDAN). NDAN patients display amyloid and tau pathology, but they resist to dementia [154]. The levels of 8-oxo-dG and 4-HNE in relation to the accumulation of neurotoxic Aβ peptide were increased in cortices of AD patients in comparison to NDAN. Further, NDAN patients show cell-specific mechanisms to counteract redox imbalance [154]. These individuals display low oxidative damage, associated with high levels of antioxidant response (SOD2, PGC1α, PPARα, CAT). PGC1α expression, as a key regulator of antioxidant response, was lower in AD and similar to control levels in NDAN individuals, while expression of miRNA-485, a PGC1α upstream regulator, was significantly increased in AD cortex as compared to the control and NDAN subjects [154]. Activation of PGC1α-mediated antioxidant pathway may prevent OS in AD [154].

Antioxidant dietary supplements are seen as a strategy to improve well-being and prevent disease. Synaptic dysfunction and synapse loss are key hallmarks of AD, present in the early stages of the disease before clinical symptoms appear [79,86,156]. Synaptic dysfunction as a therapeutic target has been studied in animal models and in clinical trials [157,158,159]. To this aim, dietary supplements containing antioxidants and phospholipids are designed to improve the synaptic and subsequently the cognitive function [160]. Dietary enrichment of nutrients that are precursors of synaptic membrane phospholipids can enhance phosphatide synthesis, the number of dendritic spines and the levels of pre- or post-synaptic proteins, which are all needed for the formation of new synapse. Preclinical studies showed that administration of specific combinations of the phospholipid synthesis-promoting nutrients can enhance neurotransmission and cognitive function [159].

This background plus the notion that circulating levels of these nutrients are lower in AD compared with healthy controls led to one of the first multicenter clinical trial to test the hypothesis that a diet supplement containing antioxidants and a combination of phospholipid synthesis-promoting nutrients could improve the synaptic function and the cognitive symptoms [160]. Souvenaid, a medical food which contains Fortasin Connect, was developed and administered to mild AD patients [160]. The study showed preliminary suggestion of continued improvement in memory performance. Recently, Tadokoro et al. introduced a novel antioxidative supplement, Twendee X (TwX). The multicenter interventional study conducted on MCI patients indicates that the antioxidant supplement TwX is beneficial for cognitive function in MCI [161]. Controlling NO overproduction with phytochemicals and nutraceuticals, and selective iNOS inhibition have been studied as potential disease-modifying-treatments in PD, AD, MS, and other inflammatory disorders [34,37,162].

Considering that a potential drug treatment for AD can counteract the biological mechanism that trigger OS, we have analyzed the current knowledge on clinical trials on AD treatment. Cummings and colleagues have reviewed www.clinicaltrials.gov (accessed on 11 August 2021) and described the pipeline of drugs in development for AD for the sixth year in a row [163]. The potential drugs have been classified based on the phase of the clinical trial, common Alzheimer’s and related dementias research ontology (CADRO) targets and mechanism of action of the drug. Looking at the clinical trials that have reached phase 3, beside the very recently approved Aducanumab, there is a progressive interest in the non-amyloid targets [163]. Indeed, some of the treatments are designed to modify the biology of the disease acting on synaptic dysfunction, mitochondrial dysfunction, microglial modulation, and reducing OS [163]. Of the 18 agents in phase 3 classified as disease-modifying treatments, three have OS as mechanism of action, two have metabolism/bioenergetic, two have antioxidant properties, and among the three agents acting on synaptic plasticity/neuroprotection, Azeliragon is also meant to ameliorate OS (Table 2). Interestingly, Blarcamesine or ANAVEX2-73 (NTC03790709) is a chemical compound known as an agonist of the sigma 1 receptor [164]. The therapeutic purpose of the treatment with this compound is its potential to correct a number of metabolic and synaptic abnormalities, as this receptor has been shown to restore cellular homeostasis and synaptic plasticity when activated by targeting mitochondrial dysfunction and associated OS, protein misfolding, proteostasis, and associated autophagy, and neuroinflammation [164]. The multinational trial using biomarker and precision medicine to monitor patients with mild AD or MCI is still ongoing. The same compound is also in development for Parkinson’s disease dementia.

Another molecule in Phase 3 is Azeliragon (NTC03980730), an inhibitor of RAGE that can be administered orally [163]. RAGE binds AGEs, products of the glycosylation of proteins. AGEs are adducts produced and slowly accumulated in the body during aging and under OS. Hyperglicemic conditions as the ones observed in diabetes can also cause the nonenzymatic glycation of proteins and the formation of AGEs. As previously discussed in this review, AGEs through the binding to the receptor, can produce ROS and be involved in neurodegeneration [42]. RAGE can also bind amyloid and is upregulated in astrocytes and microglia of AD patients [47]. The clinical trial will end in 2023.

Icosapent ethyl (NCT02719327) is a purified form of the omega-3 fatty acid, eicosapentaenoid acid, administered as a dietary supplement. It is an approved drug for the treatment of severe hypertriglyceridemia, in the current trial it is supposed to counteract OS, reducing inflammation and improving synaptic function. The same agent omega 3 (NCT03691519) is currently in another phase 3 trial, classified in OS for CADRO mechanism and for its antioxidant action [163]. DHA exerts its indirect antioxidant action modulating thioredoxin and glutathione pathways, and DHA supplementation is beneficial in improving cognitive function in mild AD [165]. However, omega 3 fatty acids are prone to peroxidation and an altered balance between cholesterol and oxysterols can reverse the beneficial effects. Thus, diet supplements with appropriate omega 3/omega 6 and low content of cholesterol can be considered a strategy in AD prevention [166].

Energy metabolism is also implicated in AD pathogenesis, and its dysregulation can be linked to OS, as discussed previously. Regarding the role of glucose metabolism in AD and the association of Type 2 Diabetes with AD, the medicament metformin (NTC04098666) is in phase 3 [163]. Metformin is an insulin sensitizing medicine, a first-line treatment and a widely prescribed oral treatment for type 2 diabetes. Glucose metabolism amelioration in CNS is supposed to improve the cognitive decline of aMCI patients or prevent the cognitive decline in obese people [163,167]. The trial will end in 2024. Tricaprilin (NCT04187547) is an oral formulation of the caprylic triglyceride, designed to improve mitochondrial and neuronal function by inducing mild ketosis.

Plants extracts with antioxidant properties as *Ginkgo Biloba* extracts are also extensively studied as dietary supplements for AD patients. *Ginkgo Biloba* (NCT03090516) extracts are supposed to improve mitochondrial function and brain blood flow, acting as a cognitive enhancer [163]. As reported, the trial is in phase 3. Regardless, discordant results, from testing this extract on the cognitive function of AD patients and healthy people, were found and reviewed by Liu et al., 2020 [168].

## 9. Conclusions and Future Perspectives

The study of accumulation of the products of oxidative damage in the brain and specimens has surely shed light on the early events of AD pathology and has contributed to the knowledge of AD pathogenesis. Aβ oligomers can induce oxidative damage to membranes of neuron synapses, mitochondria, and other brain cells, such as astrocytes and microglia. On the other hand, OS is a state which can be determined by aging and other comorbidities, representing a risk factor for AD in health people or a progression factor for the MCI patients. In this case, a systemic OS environment could be one of the components leading to neuronal damage in AD the brain.

Despite the failure of studies on oxidative damage products in AD to reveal, till now, one or more reliable marker, oxidative damage can be localized to mitochondria in AD brain and ROS may act as second messenger, initiating a downstream signaling pathway to face up the alteration in the redox balance [169]. Besides, oxidative damage may be linked to the genetic risk of AD, and in this view more relevant in patients carrying the *APOE* ε4 allele [169].

At the time being, metabolomic and proteomic blood-signature seems to be the more efficient approach to provide a complete view of the OS-mediated pathological changes caused by AD and together with the clinical biomarkers and neuropathology the more appropriate guide towards a personalized medicine in AD patients.

Regarding a potential use of metabolites related to oxidative damage as biomarkers, it has to be acknowledged that it is unlikely to find a peripheral specific marker, as many pathways related to oxidative damage occur not only in AD but in other neurodegenerative conditions. Nevertheless, the identification of a biomarker with a high negative predictive value may be useful in the context of primary care setting, i.e., a screening tool not to be intended as a diagnostic but as a gatekeeper to further confirm the diagnostic procedures (CSF biomarkers, PET with amyloid tracer) [170].

Concerning the treatment of patients with antioxidative compounds, clearly any supplementation can be considered curative, but, in the context of a multifactorial disease, it may help to reduce or limit pathogenic events eventually leading to neurodegeneration. Furthermore, the progressive interest in non-amyloid treatments could be an alternative potential approach to diseases-modifying treatments and the possibility that non-invasive and new drug delivery system could pass the BBB a future perspective in AD study.

## Figures and Tables

**Table 1:** Summary of recent studies on OS-related biomarkers for diagnosis and progression evaluation in AD.

Body Fluid	Biomarkers	Disease Stage	Method	Reference
Saliva	AGE	AD, VD, MD	Fluorescence	[26]
	AOPP, 8-isop		Colorimetric	[26]
	Lipid peroxidation compounds	AD	UPLC-MS/MS	[140]
Plasma	AGE AOPP, 8-isop, 8-OH-dG Lipid peroxidation compounds Isoprostanes	AD, VD, MD MCI AD/AD progression	Fluorescence Colorimetric UPLC-MS/MS	[26] [26] [139] [141,143]
Urine	Lipid peroxidation compounds 8-OHdG/2-dG	AD MCI/AD	UPLC-MS/MS UPLC-MS/MS	[25] [144]
	3-NO_2_-Tyr/p-Tyr			[144]
	m-Tyr/Phe, o-Tyr/Phe			[144]
Serum	Energy metabolism Mitochondrial function	AD AD	Metabolomic Proteomic	[149] [152]
CSF	Energy metabolism Sugars, Met/Tyr regulation	AD MCI	Proteomic Metabolomic	[27] [150]
Blood Blood cells	Isoprostanes, PDG2	AD	Metabolomic	[22]
	Fatty acid NO2-aLA (C18:3)			[22]
	L-arginine/NO pathway	AD, VD, MD	Metabolomic	[35]
	GR, GSH, GSSG, GSSG/GSH	AD/MCI	Fluorometric assay	[153]
Frontal cortex	8-oxo-dG, 4-HNE	AD	Immunofluorescence	[154]

Abbreviations: 3-NO_2_-Tyr/p-Tyr, 3-nitrotyrosine; 4-HNE, 4-hydroxy-2-trans-nonenal 8-isop, 8-isoprostanes; 8-OH-dG, 8-hydroxy-2-deoxyguanosine; AD, Alzheimer’s diseases; AGE, advanced glycation end products; AOPP, advanced 8-isoprostanes oxidation protein products; MD, mixed dementia; m-Tyr/Phe, meta-tyrosine; o-Tyr/Phe, orto-tyrosine; PGD2, prostaglandin2; VD, vascular dementia; UPLC/MS, liquid chromatography coupled to tandem mass spectrometry.

**Table 2:** Summary table of phase 3 clinical trials with agents related to OS mechanism of action or antioxidant properties in patients with AD.

Agent	Mechanism of Action	N. ClinicalTrial.gov	Estimated End Date
Azeliragon	RAGE antagonist	NCT03980730	July 2023
Blarcamesine (ANAVEX2-73)	Sigma 1 receptor antagonist, ameliorate OS and mitochondrial disfunction	NCT03790709	December 2021
*Ginko biloba*	Plant extracts with antioxidants properties	NCT03090516	March 2020
Icosapent ethyl	Decrease OS, improve synaptic function	NCT02719327	November 2021
Metformin	Improve CNS glucose metabolism	NCT04098666	April 2024
Omega 3 (DHA + EPA)	Antioxidant, ameliorate OS	NCT03691519	December 2023
Tricaprilin	Energy metabolism Improve mitochondrial and neuronal function inducing ketosis	NCT04187547	February 2023
